# Predicting Complexation Thermodynamic Parameters of β-Cyclodextrin with Chiral Guests by Using Swarm Intelligence and Support Vector Machines

**DOI:** 10.3390/ijms10052107

**Published:** 2009-05-14

**Authors:** Chakguy Prakasvudhisarn, Peter Wolschann, Luckhana Lawtrakul

**Affiliations:** 1 School of Technology, Shinawatra University, Shinawatra Tower III, 15th floor, 1010 Viphavadi Rangsit Road, Chatuchak, Bangkok, 10900, Thailand; E-Mail: chakguy@shinawatra.ac.th (C.P.); 2 Institute of Theoretical Chemistry, University of Vienna, Währinger Straβe 17, Vienna, 1090, Austria; E-Mail: karl.peter.wolschann@univie.ac.at (P.W.); 3 Sirindhorn International Institute of Technology (SIIT), Thammasat University, P.O.Box 22 Thammasat Rangsit Post Office, Pathum Thani, 12121, Thailand

**Keywords:** Particle Swarm Optimization, Support Vector Machines, QSPR, β-cyclodextrin inclusion complexes

## Abstract

The Particle Swarm Optimization (PSO) and Support Vector Machines (SVMs) approaches are used for predicting the thermodynamic parameters for the 1:1 inclusion complexation of chiral guests with β-cyclodextrin. A PSO is adopted for descriptor selection in the quantitative structure-property relationships (QSPR) of a dataset of 74 chiral guests due to its simplicity, speed, and consistency. The modified PSO is then combined with SVMs for its good approximating properties, to generate a QSPR model with the selected features. Linear, polynomial, and Gaussian radial basis functions are used as kernels in SVMs. All models have demonstrated an impressive performance with *R^2^* higher than 0.8.

## 1. Introduction

β-cyclodextrin (β-CD) is a cyclic oligosaccharide that naturally contains seven glucose residues linked by (1–4)-glycosidic bonds, with a hydrophilic outer surface and a relative hydrophobic central cavity, which can form complexes with appropriate guest molecules. It has received increasing attention in the pharmaceutical field for modifying drug physicochemical properties, such as solubility, stability and bio-availability, reducing their toxicity and side effects, and suppressing unpleasant taste or smell [1,2].

The high interest in the stability constants of CD-host complexes has initiated the search for proper models for predicting these association constants or the related free energies of complexation. The aim is not only to select convenient CDs for the complexation of a particular compound, but also to get some insight into the physico-chemical parameters influencing the affinity between host and guest molecules. The availability of a large amount of experimental data led to several interesting predictive models. The inclusion reactions of a series of benzene derivatives were used for a correlation model [3]. Diverse experimental information was used to develop a prediction model for the free energy of complexation using several molecular descriptors [4–7]. A CoMFA approach was applied to the binding constants of some organic compounds and various CDs [8]. An analysis of the complexation of 30 carboxylic acids and their anions was performed using a two-parameter correlation model [9]. Artificial neural networks were also used to correlate some molecular descriptors with complexation constants [10,11]. The free energies of a larger dataset of compounds complexed with all natural CDs were considered using a molecular-size based model [12,13]. Energies obtained from molecular docking of the guest molecule to the CDs’ cavities were used for systematic investigations of the interaction of organic substances with CDs [14–16]. A model correlating physico-chemical parameters directly with the solubility of the complexes was also presented [17]. A newer improved empirical model has been published just recently [18]. Most of these investigations considered the “natural” CDs, and generally, models were obtained with sufficient predictive power. The molecular descriptors are substantially different for various CDs, indicating differences in reaction mechanisms, or as a consequence of the varying flexibility of the ring system. Not much has been done up to now for the prediction of interaction energies with modified CDs, or considering different CD derivatives.

In this study, two novel approaches, Binary Particle Swarm Optimization (BPSO) [19] and Support Vector Machines (SVMs) [20] are used to predict the thermodynamic parameters for the 1:1 inclusion complexation of enantiomeric pairs of chiral guests with β-CD. SVMs represent a relatively new type of learning machine. They were designed to minimize the structural risk by minimizing an upper bound of the generalization error rather than the training error. Therefore, the over-fitting problem in machine learning is solved successfully. Another outstanding property of SVMs is that a solution obtained is always unique and globally optimal. In this paper, SVMs are investigated for a quantitative structure-property relationship (QSPR) study, to reduce the complexity of QSPR modeling and utilize the attractive properties of: not requiring the gradient information, consistent results, and fast convergence. The PSO was used to choose a set of important features.

Several factors such as number of atoms, van der Waals surface area, ionization potential, molecular weight, molar refractivity, atomic connectivity index, molecular flexibility, and angle bend energy, etc., influence thermodynamic properties. Only some of these factors strongly affect these thermodynamic properties and are controlled or set up in advance. The selection of these parameters is traditionally conducted by multiple linear regressions, partial least squares, and principle component analysis methods. Consequently, their assumptions must be verified and validated before the developed model can accurately be used. This results in a predictive model which may compromise the quality of the obtained products and/or efficiency of the modeling process.

With the increasing need for more accurate and practical evaluation QSPR models, techniques in artificial intelligence, particularly Artificial Neural Networks (ANNs), are receiving more attention in industry and academia today because they can be used to learn relationships between thermodynamic properties and their parameters. However, a number of parameters such as network topology, learning rate, and training methods have to be fine-tuned before they are deployed successfully. Furthermore, drawbacks like local optima, overfitting, and long learning time tend to occur.

Theoretically, the aforementioned shortcomings of ANNs have been countered by the development of Support Vector Machines. Unlike ANNs which minimize empirical risk, SVMs are designed to minimize the structural risk, by minimizing an upper bound of the generalization error, rather than the training error. Therefore, the overfitting problem in machine learning is solved successfully. Another outstanding property of SVMs is that the task of training SVMs is mapped to a uniquely solvable linearly constrained quadratic programming problem. This produces a solution that is always unique and globally optimal. They have been extended to solve regression problems as well.

In this paper, Support Vector Regression (SVR), which is based on Support Vector Machines, is investigated as an alternative technique for QSPR prediction. It has shown very good results for function approximation of Quantitative Structure-Activity Relationships (QSAR) [21]. The SVR retains much of the elegance of the SVMs, such as good generalization and global optimal properties, and has no normal distribution assumption requirement. The linear approximation is a fundamental concept of SVR. Its extension to a nonlinear case is achieved by using the mechanism of inner-product kernel to avoid the problem of dimensionality. To speed up its regression, the use of a proper kernel is calculated in advance. Even though this kernel computation requires large memory space, various problem optimizations have already been proposed [22,23].

Since SVM can build a very reliable QSPR model based on the training data, it is incorporated in our feature selection process. The Pearson correlation coefficient (*R*) is then treated as the objective function for a formulated optimization problem. Our previous paper [21] presented a similar approach by attempting to optimize *R* for predictive QSPR model building with a combined Feed-Forward Neural Network and a Particle Swarm Optimization. The PSO also showed good performance and was suitable for use with the found model, where no explicit relation between inputs and outputs was available. With attractive properties of no requirements for gradient information, consistent results, fast convergence, and successful applications in [24–28], PSO is then selected as an optimizer in this work.

Therefore, the purpose of this study was to develop a procedure that can determine key features for predicting complexation thermodynamic parameters of β-CD complexes with enantiomeric pairs of a chiral guest.

## 2. Methodology

The proposed methodology consists of two parts: feature selection and QSPR modeling. First, a machine learning technique called Support Vector Machines (SVMs) is used to capture characteristics of QSPR and their factors, because of the SVMs’ superior properties of generalization and global optima. They are next incorporated in an optimization problem so that a relatively new, effective, and efficient optimization algorithm, Particle Swarm Optimization (PSO), is applied to find key parameters. The cooperation between both techniques can produce a very good predictive QSPR model.

### 2.1. Support Vector Machines Based QSPR Model

SVMs represent a relatively new type of learning machine. They are an approximate implementation of the method of structural risk minimization, which attempts to minimize the generalization error, which occurs when the machines are tested with unseen data. The generalization error rate is bounded by the sum of a pair of competing terms, the training error rate and the confidence interval, which depends on the Vapnik-Chervonenkis (VC) dimension. Hence, the VC dimension and the training error (empirical risk) are both minimized at the same time. To realize this in SVMs, a structure is imposed on the set of hyperplanes, by trying to obtain the weight vector **w** having the minimum Euclidean norm. Coupled with dual transformations, the optimization model yields a global optimum. These key properties really separate the SVM from other learning machine algorithms.

In regression problems, the problem of approximating the following set of data {(**x**_1_, *y*_1_),..., (**x***_l_*, *y_l_*)} ⊂ ℜ*^n^* × ℜ with a linear function *f* (**x**) = 〈**w**, **x**〉 + *b*, where **w** ∈ ℜ*^n^*,*b* ∈ ℜ, and 〈.,.〉 represents dot product, is taken into consideration. The **x**_i_ is the set of descriptors, and y_i_ is the output, which is the thermodynamic value. The ɛ-insensitive loss function proposed by Vapnik [20] is commonly incorporated with SVMs (ɛ-SVR) to create sparseness in the support vectors and to embed the robustness of the Huber’s loss function. This means that f(**x**) is allowed to vary at most ɛ deviation from the target, and is as flat as possible, simultaneously. If the deviations are larger than the ɛ specified, this implies a bad fit and this function is proportionally penalized with constant C. This constant C determines the tradeoff between the training errors and model complexity. The flatness test of f(**x**) is accomplished by searching for the smallest **w**. Hence, a formulation of ɛ-SVR is described by:

min:

(1) $$\frac{1}{2}{\left\|w\right\|}^{2}+C\sum_{i=1}^{l}\left(\xi_{i}+\xi_{i}^{*}\right)$$

subject to:

$$\begin{array}{l} y_{i}-\langle \text{w},{\text{x}}_{i}\rangle -b\leq ɛ+\xi_{i}\\ \langle \text{w},{\text{x}}_{i}\rangle +b-y_{i}\leq ɛ+\xi_{i}^{*}\\ \xi_{i},\xi_{i}^{*}\geq 0 \end{array}$$

Everything above ɛ is captured in slack variables ξ_i_ and everything below -ɛ is captured in slack variables $\xi_{i}^{*}$. This ɛ-insensitive loss function,*|ξ|_ɛ_*is defined as:

(2). $${\left|\xi \right|}_{ɛ}=\{\begin{array}{cc} 0 & ;\text{if}\left|f(\text{x})-y\right|<ɛ\\ \left|f(\text{x})-y\right|-ɛ & ;\text{otherwise} \end{array}$$

Using the Lagrangian multipliers and the Karush-Kuhn-Tucker (KKT) conditions, the following dual problem is obtained:

(3) $$\underset{\alpha }{\text{max}}-\frac{1}{2}\sum_{i=1}^{l}\sum_{j=1}^{l}(\alpha_{i}-\alpha_{i}^{*})(\alpha_{j}-\alpha_{j}^{*})\langle {\text{x}}_{i},{\text{x}}_{j}\rangle -ɛ\sum_{i=1}^{l}(\alpha_{i}+\alpha_{i}^{*})+\sum_{i=1}^{l}y_{i}(\alpha_{i}-\alpha_{i}^{*})$$

subject to:

$$\sum_{i=1}^{l}(\alpha_{i}-\alpha_{i}^{*})=0$$

and:

$$\alpha_{i},\alpha_{i}^{*}\in [0,\;C],\;\text{i}=1,\ldots ,1\text{.}$$

Transforming into dual form yields a quadratic programming problem with linear constraints and a positive definite Hessian matrix. This leads to a global optimum. A nonlinear form is usually required to adequately model data. Hence, a nonlinear mapping, *ϕ*, is used to map data from an input space into a higher dimensional intermediate space, where linear regression is performed. Consequently, complications result in the complexity of *ϕ* and the problem of dimensionality in Equation (3). To alleviate these difficulties, the inner-product kernel is then introduced as follows:

$$K({\text{x}}_{i},{\text{x}}_{j})=\langle \phi ({\text{x}}_{i}),\phi ({\text{x}}_{j})\rangle .$$

The dimensionality of the intermediate space is thus hidden from the remaining computations. Some of the most widely used kernels, such as linear, polynomial, and Gaussian radial basis functions (RBF), were tested in this study. The kernel function is employed in the optimization models above by replacing 〈.,.〉 with *K*(.,.). This adds the capability to approximate both linear and nonlinear functions.

In summary, the main advantages of SVR are implicit mapping by using kernels in handling nonlinear data, convexity of quadratic optimization, and generalization properties. In addition, distribution of the data is not necessarily assumed in advance, which makes it very promising for real-world problems.

### 2.2. Particle Swarm Optimization

PSO was introduced by Kennedy and Eberhart [19] to imitate social behavior of animals such as birds flocking in searching for food. Each particle flies in hyperspace searching for the best solution by adjusting position and velocity based on its own flying experience (pbest) and its companions’ experience (gbest). The inertia weight *w* was later introduced to improve the PSO optimizer. It is very attractive because the requirements of gradient information are not needed. Hence, it is unaffected by discontinuities of the objective function. The equations used consist of flexible and well-balanced mechanisms to enhance the global and local exploration abilities. These allow a thorough search and simultaneously avoid premature convergence. In addition, PSO uses probabilistic rules for a particle’s movements. Therefore, it is quite robust for local optima. The standard PSO consists of the following steps [19,29]:

1. Initialize a population of I particles with random positions and velocities in D dimensions.
2. Evaluate the desired optimization function in D variables for each particle.
3. Compare the evaluation with the particle’s previous best value, pbest[i]. If the current value is better than pbest[i], then pbest[i] = current value and the pbest location, pbestx[i][d], is set to the current location in d-dimensional space.
4. Compare the evaluation with the swarm’s previous best value, (pbest[gbest]). If the current value is better than pbest[gbest]), then gbest = current particle’s array index.
5. Change the velocity and position of the particle according to the following equations, respectively:
  (4), $$\begin{array}{ll} \text{V}[\text{i}][\text{d}]= & \text{V}[\text{i}][\text{d}]\;+\;{\text{c}}_{1}*\text{rand}()*(\text{pbestx}[\text{i}][\text{d}]\;-\text{ presentx}[\text{i}][\text{d}])\;+\\ & {\text{c}}_{2}*\text{rand}()*(\text{pbestx}[\text{gbest}][\text{d}]\;-\text{ persentx}[\text{i}][\text{d}]) \end{array}$$
  (5). presentx[i][d] = presentx[i][d] + V[i][d]
6. Loop to step 2 until a stopping criterion, a sufficiently good evaluation function value, or a maximum number of iterations, is met.

In feature selection, the input presented to the regression modeling is in the form of a table where the rows represent chemical compounds and the columns are the molecular descriptors. Each compound contains a value for each corresponding factor. How accurately a QSPR model can predict the biological activity of the compounds depends on their values in a subset of the selected features. Hence, the selection of each column or feature is treated as a binary number. A numerical value of zero is used to represent that the corresponding descriptor is not selected for QSPR modeling. Otherwise, a numerical value of one is assigned. This binary problem calls for some modification of the original PSO. Thus presentx[i][d], which represents the value stored by the i^th^ particle in the d^th^ dimension, can only take on a binary value, instead of a real valued number. This indicates whether the d^th^ feature is selected or not. Note that the D dimensions above are equal to the total number of descriptors. After the update step (Equation (5)), presentx[i][d] is discretized to a binary value by using probabilistic selection or roulette wheel selection. The fractional values of presentx[i][d] are treated as probability thresholds to determine subset membership. Each dimension or feature of the particle is assigned a slice of a roulette wheel whose size is proportional to presentx[i][d]. The subset is assembled by spinning the wheel and selecting the features to which the wheel’s marker points. This process is repeated k times, which are the predefined number of selected features. The chosen descriptors are then set to 1 and the remaining parameters are set to 0. The actual probabilities, p_id_, are computed as follows:

(6) $$p_{id}=\frac{x_{id}^{a}}{\sum_{d=1}^{D}x_{id}^{a}}$$

where x_id_ is the fractional coordinates presentx[i][d] after the update step (Equation (5)), and *a* is a scaling factor or selection pressure and is set to 2. This binary PSO (BPSO) still presents the same advantages as the original PSO. The near-optimal solutions are found much faster, compared with the performance of a random search or an exhaustive search. This allows BPSO to perform feature selection efficiently in datasets with large numbers of descriptors. The objective function evaluated by the BPSO is the Pearson correlation coefficient that measures the quality of QSPR model with the selected features:

(7) $$R=\frac{N\sum_{n=1}^{N}y_{i}{\hat{y}}_{i}-\sum_{n=1}^{N}y_{i}\sum_{n=1}^{N}{\hat{y}}_{i}}{\sqrt{\left[N\sum_{n=1}^{N}y_{i}^{2}-{\left(\sum_{n=1}^{N}y_{i}\right)}^{2}][N\sum_{n=1}^{N}{\hat{y}}_{i}^{2}-{\left(\sum_{n=1}^{N}{\hat{y}}_{i}\right)}^{2}\right]}}$$

where *N* is the number of training compounds for regression and *y_i_* and *ŷ_i_* are the measured and the predicted activities of the i^th^ compound, respectively.

### 2.3. Chiral Guest Dataset and Descriptor Generation

The complex stability constant (ln *K*), the standard free energy (Δ*G*°), the enthalpy (Δ*H*°) and the entropy change (*T*Δ*S*°) for the 1:1 inclusion complexation of enantiomer pairs of 74 selected chiral compounds with β-CD were taken from the experiments of Rekharsky and Inoue [30]. The values are given in Table 1. The guest structures were constructed by using the HyperChem program and fully geometrically optimized at the HF/3–21G level by the Gaussian03 program [31]. Two hundred structural properties were calculated by the Molecular Operating Environment (MOE) program package [32]. Descriptors are categorized by class: 2D descriptors (2D) are calculated from purely atomic and connectivity properties, internal 3D descriptors (i3D) use 3D coordinate information about each molecule, and external 3D descriptors (x3D) use absolute 3D atomic coordinate information, but also require an absolute frame of reference (such as, the molecules docked into the same receptor). The chiral guest dataset consisted of 56 compounds for training the models and 18 compounds for testing the quality of models.

### 2.4. QSPR Models

The PSO was adopted for major descriptor selection in QSPR of the chiral guest dataset. Swarm parameters are 50 particles and 100 iterations. The iterative PSO attempts to select the key features that maximize the Pearson correlation coefficient (*R*), resulting in a QSPR model developed by SVMs. The linear approximation is a fundamental concept of SVMs. Some of the most widely used kernels, such as linear, polynomial, and Gaussian radial basis functions (RBF) were tested in this study. This adds the capability to approximate both linear and nonlinear functions. Both PSO and SVMs were implemented in MATLAB 7.0.4 running on a Pentium IV (2.4 GHz) computer. The correlation coefficient for all PSO-SVM models are the average values from 10 calculations.

## 3. Results and Discussion

Table 2 shows that the PSO-SVMs with three different kernels give very good results. All models have demonstrated an impressive performance with *R^2^* for training set (*R^2^_Training_*) higher than 0.8. The nonlinear kernels give better results than the linear function for the training chiral guest dataset. The polynomial kernel, with *R^2^_Training_* between 0.9991 and 0.9994, has better calibration correlation coefficients than the Gaussian RBF kernel, whereas the Gaussian RBF gives much better predictions than those obtained with the polynomial SVM. These agree with our previous work [33], in which K and ΔH° of the given enantiomer pair dataset were predicted best with a Gaussian RBF kernel.

The numbers of descriptors in the QSPR models of these thermodynamic properties are further investigated by using the Gaussian RBF kernel, which gives the best outcome for the chiral guest dataset. The statistics for all PSO-SVM models are given in Table 3. Models with four features also provide satisfactory predictive abilities when compared to the eight feature models. The best prediction performances of the individual models are presented in Table 4.

**Figure 1.**
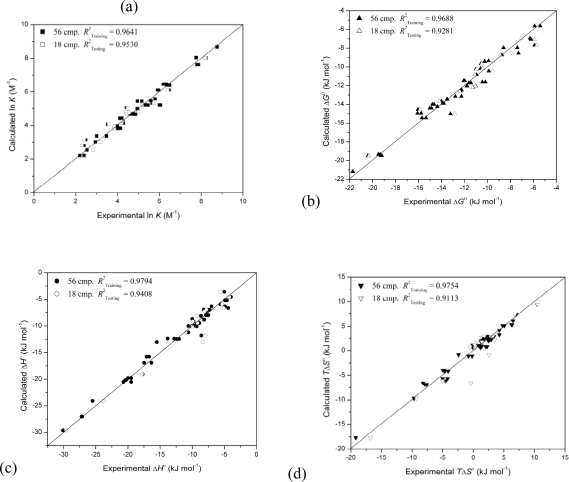
Plots of calculated thermodynamic parameters by PSO-SVM with Gaussian RBF kernel models with 4 features, *versus* the experimental values: (a) complex stability constant (ln *K*), (b) standard free energy (Δ*G*°), (c) enthalpy (Δ*H*°) and (d) entropy change (*T*Δ*S*°).

Table 5 presents the selected descriptors in the best prediction QSPR models. All models were developed with four 2D descriptors, which use the atoms and connection information of the molecule for the calculation. This illustrates that the molecular size and shape factors are important for the thermodynamic properties of 74 chiral compounds in 1:1 inclusion complex with β-CD. The plots of the best QSPR models with 4 features for ln *K*, Δ*G*°, Δ*H*° and *T*Δ*S*° by PSO-SVM integration against the experimental values are shown in Figure 1.

Even though PSO-SVM methods are not able to explain the values of descriptors in the models, the maximum outlier from the QSPR models can point out the error of the experimental data. In Δ*H*° and *T*Δ*S*° predictions by four features, PSO-SVM models indicate that cmp. 32 is the maximum outlier. Considering the values in Table 1, experimental results show that Δ*H*° of cmp. 31 (1*R*, 3*S*-camphoric acid) and cmp. 32 (1*S*, 3*R*-camphoric acid) are −15.5 and −8.3 kJ mol^−1^, and *T*Δ*S*° are −8.3 and 0.4 kJ mol^−1^, respectively. The experimental results have different values for this enantiomeric pair, whereas the PSO-SVM models have identical results: Δ*H*° = −13.02 kJ mol^−1^and *T*Δ*S*° = −6.63 kJ mol^−1^.

## 4. Conclusions

This work demonstrated that the combination of PSO and SVMs can be applied to effectively and efficiently select major features in QSPR modeling of the thermodynamic parameters of 1:1 inclusion complexation of enantiomeric pairs of chiral guests with β-CD. This responds to the needs of drug designers for prediction of the thermodynamic parameters of new compounds in complexation with β-CD. The method was based on a discrete binary modification of PSO. The fitness function was the Pearson correlation which was curve fitted by SVMs. The modified PSO appeared to be an effective and efficient algorithm, which robustly finds near-optimal and consistent results with short computer code and simple mathematical operators, while converging rather quickly. The SVMs showed excellent performance in predicting ln *K*, Δ*G*°, Δ*H*° and *T*Δ*S*°, by considering major selected features. The combination of the adopted methods showed satisfactory results with the large dataset.

## Figures and Tables

**Table 1.** Experimental Thermodynamic parameters: ln *K* (M^−1^), Δ*G*° (kJ mol^−1^), Δ*H*° (kJ mol^−1^) and *T*Δ*S*° (kJ mol^−1^) of 74 chiral compounds in 1:1 inclusion complexation with β-CD taken from Ref. [30] and the values from the best prediction QSPR models with four features.

**cmp**	**guest**	**Experimental**				**Calculation***^b^*			

		**ln *K***	**Δ*G°***	**Δ*H°***	***T*Δ*S°***	**ln *K***	**Δ*G°***	**Δ*H°***	***T*Δ*S°***
1	*N-*acetyl-D-phenylalanine	4.11	−10.18	−8.14	2.04	4.43	−11.61	−8.82	0.78
2	*N-*acetyl-L-phenylalanine	4.21	−10.44	−8.17	2.27	4.43	−11.61	−8.82	0.78
3	*N-*acetyl-D-tryptophan	2.54	−6.30	−25.50	−19.20	2.54	−6.94	−24.09	−17.72
4*^a^*	*N-*acetyl-L-tryptophan	2.84	−7.04	−23.80	−16.80	2.54	−6.94	−24.09	−17.72
5	*N-*acetyl-D-tyrosine	4.83	−11.97	−16.70	−4.70	4.65	−11.48	−15.77	−3.92
6	*N-*acetyl-L-tyrosine	4.87	−12.07	−17.10	−5.00	4.65	−11.48	−15.77	−3.92
7	(1*R*,2*S*)-2-amino-1,2-diphenylethanol	4.01	−9.90	−10.00	−0.10	3.84	−9.35	−10.11	0.65
8*^a^*	(1*S*,2*R*)-2-amino-1,2-diphenylethanol	3.83	−9.50	−10.00	−0.50	3.84	−9.35	−10.11	0.65
9	(*R*)-benzyl glycidyl ether	5.46	−13.52	−9.20	4.30	5.21	−13.45	−9.58	4.22
10	(*S*)-benzyl glycidyl ether	5.43	−13.50	−9.30	4.20	5.21	−13.45	−9.58	4.22
11	2,3-*O*-benzylidene-D-threitol	4.76	−11.81	−7.56	4.25	4.70	−12.05	−7.98	3.30
12	2,3-*O*-benzylidene-L-threitol	4.74	−11.76	−7.49	4.27	4.70	−12.05	−7.98	3.30
13*^a^*	(2*R*,3*R*)-3-benzyloxy-1,2,4-butanetriol	4.42	−10.95	−8.07	2.90	4.79	−10.23	−7.23	2.90
14	(2*S*,3*S*)-3-benzyloxy-1,2,4-butanetriol	4.44	−11.01	−7.79	3.20	4.79	−10.23	−7.23	2.90
15	*O*-benzyl-D-serine	4.26	−10.57	−8.90	1.70	4.25	−9.67	−9.46	2.74
16*^a^*	*O*-benzyl-L-serine	4.23	−10.50	−9.20	1.30	4.25	−9.67	−9.46	2.74
17	*N-t-*Boc-D-alanine	5.97	−14.80	−9.70	5.10	6.11	−14.00	−9.94	5.16
18	*N-t-*Boc-L-alanine	5.91	−14.64	−9.80	4.80	6.11	−14.00	−9.94	5.16
19	*N-t-*Boc-D-alanine methyl ester	6.49	−16.09	−13.82	2.30	6.40	−14.95	−12.37	2.43
20	*N-t-*Boc-L-alanine methyl ester	6.36	−15.77	−12.80	3.00	6.40	−14.95	−12.37	2.43
21*^a^*	*N-t-*Boc-D-serine	5.72	−14.19	−11.00	3.20	5.33	−13.67	−11.20	2.95
22	*N-t-*Boc-L-serine	5.65	−14.01	−10.60	3.40	5.33	−13.67	−11.20	2.95
23	(*R*)-3-bromo-8-camphorsulfonic acid	8.23	−20.41	−30.10	−9.70	8.03	−19.49	−29.63	−9.67
24*^a^*	*(S)*-3-bromo-8-camphorsulfonic acid	8.20	−20.32	−29.60	−9.30	8.03	−19.49	−29.63	−9.67
25	(*R*)-3-bromo-2-methyl-1 propanol	4.96	−12.29	−9.30	3.00	4.97	−12.47	−10.07	2.65
26	(*S*)-3-bromo-2-methyl-1 propanol	4.94	−12.25	−10.10	2.20	4.97	−12.47	−10.07	2.65
27	(*R*)-3-bromo-2-methylpropionic acid methyl ester	5.58	−13.80	−12.05	1.80	5.50	−13.90	−12.45	2.40
28	(*S*)-3-bromo-2-methylpropionic acid methyl ester	5.60	−13.90	−12.40	1.50	5.50	−13.90	−12.45	2.40
29*^a^*	(*R*)-camphanic acid	5.18	−12.85	−17.80	−5.00	5.23	−15.01	−19.07	−6.17
30	(*S*)-camphanic acid	5.33	−13.22	−17.70	−4.50	5.23	−15.01	−19.07	−6.17
31	(1*R*,3*S*)-camphoric acid	2.94	−7.30	−15.50	−8.20	3.02	−8.52	−13.02	−6.63
32*^a^*	(1*S*,3*R*)-camphoric acid	3.18	−7.90	−8.30	−0.40	3.02	−8.52	−13.02	−6.63
33	(*R*)-camphorquinone-3-oxime	7.87	−19.50	−27.10	−7.60	7.62	−19.33	−27.04	−6.91
34	(*S*)-camphorquinone-3-oxime	7.80	−19.34	−27.20	−7.90	7.62	−19.33	−27.04	−6.91
35	(*R*)-10-camphorsulfonic acid	6.34	−15.70	−20.70	−5.00	6.46	−15.44	−20.53	−5.74
36	(*S*)-10-camphorsulfonic acid	6.19	−15.35	−19.50	−4.20	6.46	−15.44	−20.53	−5.74
37*^a^*	*N-*Cbz-D-alanine	5.00	−12.40	−8.90	3.50	4.81	−12.64	−8.69	2.17
38	*N-*Cbz-L-alanine	4.99	−12.37	−10.00	2.40	4.81	−12.64	−8.69	2.17
39	(1*R*,2*R*)-*trans*-1,2-cyclohexanediol	4.44	−11.01	−3.98	7.03	4.66	−12.04	−4.54	7.43
40*^a^*	(1*S*,2*S*)-*trans*-1,2-cyclohexanediol	4.45	−11.04	−4.21	6.83	4.66	−12.04	−4.54	7.43
41	(*R*)-1-cyclohexylethylamine	5.80	−14.37	−7.85	6.50	5.59	−14.24	−7.94	6.15
42	(*S*)-1-cyclohexylethylamine	5.79	−14.36	−7.87	6.50	5.59	−14.24	−7.94	6.15
43	*O*,*O*'-dibenzoyl-D-tartaric acid	3.47	−8.60	−7.00	1.60	3.39	−7.95	−6.32	2.02
44	*O*,*O*'-dibenzoyl-L-tartaric acid	3.00	−7.40	−4.90	2.50	3.39	−7.95	−6.32	2.02
45*^a^*	Gly-D-Phe	3.85	−9.54	−7.93	1.60	3.96	−10.44	−11.79	1.74
46	Gly-L-Phe	3.99	−9.89	−8.59	1.30	3.96	−10.44	−11.79	1.74
47	(*R*)-hexahydromandelic acid	6.47	−16.05	−5.61	10.44	6.11	−14.54	−5.92	9.37
48*^a^*	(*S*)-hexahydromandelic acid	6.40	−15.87	−5.36	10.51	6.11	−14.54	−5.92	9.37
49	(1*R*,2*R*,5*R*)-2-hydroxy-3-pipanone	7.77	−19.30	−19.50	−0.20	8.05	−19.47	−19.79	−1.12
50	(1*S*,2*S*,5*S*)-2-hydroxy-3-pipanone	7.75	−19.20	−20.00	−0.80	8.05	−19.47	−19.79	−1.12
51	(*R*)-mandelic acid	2.40	−5.90	−4.90	1.00	2.20	−5.63	−5.17	1.11
52	(*S*)-mandelic acid	2.20	−5.40	−4.60	0.80	2.20	−5.63	−5.17	1.11
53*^a^*	(*R*)-mandelic acid methyl ester	4.20	−10.42	−7.80	2.60	4.13	−10.17	−6.94	−0.86
54	(*S*)-mandelic acid methyl ester	4.28	−10.60	−8.20	−2.40	4.13	−10.17	−6.94	−0.86
55	(*R*)-α-methoxyphenylacetic acid	2.40	−5.90	−4.40	1.50	2.79	−7.68	−6.63	1.75
56*^a^*	(*S*)-α-methoxyphenylacetic acid	2.30	−5.70	−5.10	0.60	2.79	−7.68	−6.63	1.75
57	(*R*)-α-methoxy-α-trifluoromethylphenylacetic acid	5.16	−12.80	−17.48	−4.70	5.45	−13.14	−16.92	−4.19
58	(*S*)-α-methoxy-α-trifluoromethylphenylacetic acid	4.95	−12.27	−16.35	−4.10	5.45	−13.14	−16.92	−4.19
59	D-phenylalanine amide	4.62	−11.44	−10.00	1.40	4.66	−11.71	−10.01	0.87
60	L-phenylalanine amide	4.69	−11.63	−10.60	1.00	4.66	−11.71	−10.01	0.87
61*^a^*	D-phenylalanine methyl ester	2.40	−5.90	−5.60	0.30	3.16	−7.07	−3.56	0.58
62	L-phenylalanine methyl ester	2.48	−6.20	−5.00	1.20	3.16	−7.07	−3.56	0.58
63	(*R*)-2-phenylbutyric acid	4.54	−11.26	−9.79	1.50	4.77	−12.20	−9.15	1.81
64*^a^*	(*S*)-2-phenylbutyric acid	4.55	−11.29	−9.91	1.40	4.77	−12.20	−9.15	1.81
65	(*R*)-3-phenylbutyric acid	6.00	−14.86	−8.62	6.24	5.22	−14.41	−8.72	5.34
66	(*S*)-3-phenylbutyric acid	6.06	−15.03	−8.68	6.35	5.22	−14.41	−8.72	5.34
67	(*R*)-1-phenyl-1,2-ethanediol	4.13	−10.23	−7.54	2.69	3.85	−9.42	−6.96	2.24
68	(*S*)-1-phenyl-1,2-ethanediol	4.14	−10.26	−7.30	2.96	3.85	−9.42	−6.96	2.24
69*^a^*	(*R*)-phenyllactic acid	4.48	−11.10	−9.34	1.80	5.06	−10.83	−8.09	2.99
70	(*S*)-phenyllactic acid	4.42	−10.95	−8.65	2.30	5.06	−10.83	−8.09	2.99
71	(*R*)-2-phenylpropionic acid	3.53	−8.74	−8.81	−0.10	4.06	−8.72	−8.84	1.18
72*^a^*	(*S*)-2-phenylpropionic acid	3.58	−8.88	−8.69	0.20	4.06	−8.72	−8.84	1.18
73	(1*R*,2*R*,3*S*,5*R*)-pinanediol	8.77	−21.74	−20.40	1.30	8.67	−21.18	−20.19	2.03
74	(1*S*,2*S*,3*R*,5*S*)-pinanediol	8.76	−21.71	−20.30	1.40	8.67	−21.18	−20.19	2.03

^a^ Compounds in test set;

^b^ The descriptors in the QSPR models are provided in Table 4.

**Table 2.** The average predictive ability of PSO-SVMs QSPR models with 8 descriptors.

***SVMs***	**ln *K***		**Δ*G*°**		**Δ*H*°**		***T*Δ*S*°**	

	***R^2^_Training_***	***R^2^_Testing_***	***R^2^_Training_***	***R^2^_Testing_***	***R^2^_Training_***	***R^2^_Testing_***	***R^2^_Training_***	***R^2^_Testing_***
**Linear**	0.8201	0.6666	0.8239	0.6349	0.9048	0.8455	0.8220	0.8257
**Polynomial**	0.9993	0.7358	0.9994	0.8213	0.9992	0.8432	0.9991	0.8251
**Gaussian RBF**	0.9983	0.9762	0.9987	0.9713	0.9983	0.9350	0.9986	0.8853

**Table 3.** The average predictive ability of PSO-SVM with Gaussian RBF kernel models.

**Number of descriptors**	**ln *K***		**Δ*G*°**		**Δ*H*°**		***T*Δ*S*°**	

	***R^2^_Training_***	***R^2^_Testing_***	***R^2^_Training_***	***R^2^_Testing_***	***R^2^_Training_***	***R^2^_Testing_***	***R^2^_Training_***	***R^2^_Testing_***
8	0.9983	0.9762	0.9987	0.9713	0.9983	0.9350	0.9986	0.8853
7	0.9977	0.9534	0.9981	0.9778	0.9978	0.9325	0.9981	0.8936
6	0.9963	0.9629	0.9967	0.9039	0.9966	0.9271	0.9967	0.8868
5	0.9869	0.9292	0.9872	0.9507	0.9932	0.9142	0.9919	0.8812
4	0.9534	0.9020	0.9496	0.8498	0.9820	0.8563	0.9572	0.8707

**Table 4.** Descriptors in the best predictive ability of PSO-SVM with Gaussian RBF kernel models.

	**Number of descriptors**	***R^2^_Training_***	***R^2^_Testing_***	**Descriptors in the model**							

				**I**	**II**	**III**	**IV**	**V**	**VI**	**VII**	**VIII**
**ln *K***	8	0.9982	0.9903	2	20	31	76	80	143	164	185
	7	0.9982	0.9922	2	12	90	94	122	144	164	
	6	0.9968	0.9879	3	20	30	94	140	167		
	5	0.9929	0.9829	3	20	27	59	134			
	4	0.9641	0.9530	12	79	114	134				
**Δ*G*°**	8	0.9985	0.9924	3	11	28	79	94	112	136	144
	7	0.9978	0.9928	2	9	111	123	129	133	140	
	6	0.9969	0.9894	3	12	94	124	133	140		
	5	0.9935	0.9849	3	20	27	59	134			
	4	0.9688	0.9281	20	72	94	122				
**Δ*H*°**	8	0.9987	0.9510	28	54	76	83	124	173	181	186
	7	0.9977	0.9385	20	79	91	140	143	153	187	
	6	0.9965	0.9417	2	21	27	112	154	176		
	5	0.9943	0.9374	16	36	79	91	122			
	4	0.9794	0.9408	20	21	30	36				
***T*Δ*S*°**	8	0.9986	0.8949	3	23	36	44	94	112	114	159
	7	0.9982	0.9371	9	20	33	100	122	158	199	
	6	0.9952	0.8991	9	12	93	94	154	164		
	5	0.9868	0.9079	6	30	39	76	90			
	4	0.9754	0.9113	8	76	114	129				

**Table 5.** The selected descriptors in four feature QSPR models.

**No.**	**Class**	**Description**
8	2D	Weiner polarity number
12	2D	PEOE Charge BCUT (3/3)
20	2D	Molar Refractivity BCUT (3/3)
21	2D	PEOE Charge GCUT (0/3)
30	2D	Molar Refractivity GCUT (1/3)
36	2D	Atom information content (mean)
50	2D	Number of chiral centers
72	2D	Total positive partial charge
76	2D	Total positive 0 van der Waals surface area
79	2D	Total positive 3 van der Waals surface area
94	2D	Fractional positive van der Waals surface area
114	2D	Third alpha modified shape index
122	2D	Number of H-bond donor atoms
129	2D	van der Waals polar surface area
134	2D	Bin 3 SlogP_(0.00, 0.10]
